# Accelerated aging of skeletal muscle and the immune system in patients with chronic liver disease

**DOI:** 10.1038/s12276-024-01287-y

**Published:** 2024-07-18

**Authors:** Thomas Nicholson, Amritpal Dhaliwal, Jonathan I. Quinlan, Sophie L. Allen, Felicity R. Williams, Jon Hazeldine, Kirsty C. McGee, Jack Sullivan, Leigh Breen, Ahmed M. Elsharkawy, Matthew J. Armstrong, Simon W. Jones, Carolyn A. Greig, Janet M. Lord

**Affiliations:** 1 NIHR Birmingham Biomedical Research Centre, University Hospital Birmingham and University of Birmingham, Birmingham, UK; 2 Institute of Inflammation and Ageing, University of Birmingham, Birmingham, UK; 3 MRC-Versus Arthritis Centre for Musculoskeletal Ageing Research, Birmingham, UK; 4 School of Sport, Exercise and Rehabilitation Science, University of Birmingham, Birmingham, UK; 5 Liver Transplantation Unit, Queen Elizabeth Hospital, Birmingham, UK

**Keywords:** Senescence, Chronic inflammation

## Abstract

Patients with chronic liver disease (CLD) often present with significant frailty, sarcopenia, and impaired immune function. However, the mechanisms driving the development of these age-related phenotypes are not fully understood. To determine whether accelerated biological aging may play a role in CLD, epigenetic, transcriptomic, and phenotypic assessments were performed on the skeletal muscle tissue and immune cells of CLD patients and age-matched healthy controls. Accelerated biological aging of the skeletal muscle tissue of CLD patients was detected, as evidenced by an increase in epigenetic age compared with chronological age (mean +2.2 ± 4.8 years compared with healthy controls at −3.0 ± 3.2 years, *p* = 0.0001). Considering disease etiology, age acceleration was significantly greater in both the alcohol-related (ArLD) (*p* = 0.01) and nonalcoholic fatty liver disease (NAFLD) (*p* = 0.0026) subgroups than in the healthy control subgroup, with no age acceleration observed in the immune-mediated subgroup or healthy control subgroup (*p* = 0.3). The skeletal muscle transcriptome was also enriched for genes associated with cellular senescence. Similarly, blood cell epigenetic age was significantly greater than that in control individuals, as calculated using the PhenoAge (*p* < 0.0001), DunedinPACE (*p* < 0.0001), or Hannum (*p* = 0.01) epigenetic clocks, with no difference using the Horvath clock. Analysis of the IMM-Age score indicated a prematurely aged immune phenotype in CLD patients that was 2-fold greater than that observed in age-matched healthy controls (*p* < 0.0001). These findings suggested that accelerated cellular aging may contribute to a phenotype associated with advanced age in CLD patients. Therefore, therapeutic interventions to reduce biological aging in CLD patients may improve health outcomes.

## Skeletal Muscle Ageing in CLD: Implications for Therapeutic Interventions

Chronic liver disease, a long-term condition damaging the liver, is causing more deaths worldwide. Patients often develop immune dysfunction and sarcopenia. This study aimed to see if CLD patients show signs of fast biological ageing, particularly in muscles and the immune system. The research compared CLD patients to healthy people, looking at muscle samples, blood samples, and immune cells to check for ageing signs. Results showed that CLD patients have faster biological ageing in muscles and immune cells, with increased epigenetic age and more senescence-associated genes. This suggests that CLD speeds up the ageing process, which could explain the common occurrence of sarcopenia and immune dysfunction in these patients. Future implications include the possibility of developing treatments targeting the ageing process in CLD patients, offering hope for better health and quality of life

This summary was initially drafted using artificial intelligence, then revised and fact-checked by the author.

## Introduction

Chronic liver disease (CLD) is now one of the leading causes of death worldwide, and the incidence of this disease has increased annually since 2009^1^. CLD patients often develop conditions normally associated with advancing age, including immune dysfunction and sarcopenia, the latter of which is present in up to 70% of patients and is characterized by loss of skeletal muscle mass, strength, and functional ability^2^. These conditions significantly increase the risk of infection, hospital admissions, risk of falls, and ultimately premature mortality^3^. However, the mechanisms driving the development of these age-related phenotypes in patients with CLD are not fully understood.

As both immune dysfunction and sarcopenia are more prevalent with advancing age^4,5^, one possible underlying mechanism contributing to the development of these conditions in CLD patients is the acceleration of the biological aging process. The core processes driving the aged phenotype are now better understood, with 12 mechanisms suggested as the hallmarks of aging, including telomere shortening, cellular senescence, mitochondrial dysfunction, altered nutrient sensing, stem cell exhaustion, and inflammation^6^. Several of these hallmarks have been identified as being associated with immune dysfunction and sarcopenia. Age-related immune dysfunction, termed immunosenescence, is characterized by telomere shortening^7^, reduced autophagy^8^, and increased T cell senescence with an associated proinflammatory secretome^9^. With respect to sarcopenia, some of the hallmarks associated with reduced muscle mass are stem cell exhaustion^10^, cellular senescence^11^, increased inflammation^12^, and reduced anabolic responses to amino acids and physical activity^13^.

The identification and validation of biomarkers of biological aging, either in an individual overall or in specific body systems, has significant clinical potential, facilitating the development and utility of personalized, targeted therapies to reduce biological aging and improve health outcomes in patients with accelerated aging as a result of chronic diseases^14^, such as CLD. Recently, an integrated score for the degree of immunosenescence, IMM-AGE, was developed based on the frequency of 20 different immune cell types^14^. The IMM-AGE score is associated with increased mortality^14,15^ and is thus an additional biomarker of biological age. Furthermore, the development of DNA methylation-based epigenetic ‘clocks’, which utilize specific regions of the DNA methylome (CpG sites), has demonstrated a strong correlation between the degree of methylation at these sites and an individual’s chronological age^16^. Importantly, an individual’s epigenetic age can diverge from their chronological age, indicating that they are aging at either an accelerated or decelerated rate^17,18^. The acceleration of epigenetic age is associated with increased disease risk and ultimately all-cause mortality^19^. Individuals with a lower epigenetic age than their chronological age typically have a longer healthspan and lifespan^20,21^. Supporting these epigenetic clocks is the recently developed DunedinPACE epigenetic clock, which, by predicting the rate of biological aging, can be used to provide insights into future health trajectories^22^.

Despite these advances and the acknowledgment of inflammation as a hallmark of aging^6^, there is a paucity of data investigating the impact of chronic inflammatory diseases, such as CLD, on the biological aging process. Biological age may provide a novel biomarker to identify CLD patients at greatest risk of disease progression and thus facilitate early intervention therapeutic strategies to prevent associated complications (i.e., ascites, hepatocellular carcinoma, variceal hemorrhage, and death) and extend healthspan. Therefore, the present study aimed to identify whether patients with CLD display accelerated biological aging phenotypes across the skeletal muscle and immune system, utilizing epigenetic, transcriptomic, and phenotypic approaches.

## Materials and methods

### Participants and ethical approval

CLD patients enrolled in the present study were recruited for a larger prospective observational study, namely, the Evaluation of Sarcopenia in Inflammatory Disease (clinical trial ID: NCT04734496), in which the inclusion and exclusion criteria have previously been reported^23^. All CLD patients had end-stage liver disease (Child‒Pugh B/C). Age- and sex-matched control participants had normal liver test results (including blood, risk factor, and imaging data), had no medical comorbidities, and were not taking any medication. Ethical approval was obtained through the Health Research Authority—West Midlands Solihull Research Ethics Committee Authority (REC reference: 18/WM/0167) for the recruitment of patients with CLD and the local Ethics Committee at the University of Birmingham (ERN_19-0831) for the recruitment of healthy controls. The present study was conducted in accordance with the Declaration of Helsinki, and all participants provided written informed consent.

### Study design

All participants reported to the laboratory in a fasted state (from 06:00 on the day of visit) and were asked to refrain from the consumption of caffeine on the morning of the trial. In addition, participants were asked to refrain from strenuous exercise for 24 h prior to their laboratory visit. Upon arrival, a fasted venous blood sample was obtained for the isolation of peripheral blood mononuclear cells (PBMCs) and serum collection. Participants underwent assessments of basic body composition, skeletal muscle mass, and functional strength as previously described^23^. A skeletal muscle biopsy was obtained from the *vastus lateralis* of the dominant leg using a Bergström needle^24^ and immediately snap frozen in liquid nitrogen. The samples were stored at −80 °C until analysis. Quadricep muscle performance tests^23^ were performed postbiopsy to ensure that the biopsy samples were obtained at rest. Measures of quadricep muscle mass and intramuscular adipose tissue (IMAT) accumulation were determined from MR images as described previously^23^.

### Blood sample processing

PBMCs were isolated by density centrifugation using Ficoll-Paque™ PLUS (GE Healthcare, UK) as previously described^25^. The isolated PBMCs were resuspended in freezing medium (10% DMSO in heat-inactivated fetal bovine serum (FBS); Sigma Aldrich, UK) and stored at −80 °C until analysis. To obtain serum, blood samples, following a 30-min incubation at room temperature, were centrifuged at 1620×*g* for 10 min. The upper serum layer was collected and stored at −80 °C until thawing on ice for analysis. Freeze‒thaw cycles were avoided.

### DNA methylation analysis and calculation of epigenetic age

DNA was isolated from muscle tissue biopsies (~10 mg) and PBMCs utilizing a commercially available DNA extraction kit (DNeasy Blood & Tissue Kit, Qiagen, Manchester, UK) following the manufacturer’s protocol. Muscle tissue was first homogenized in ATL lysis buffer using a Qiagen Tissue Ruptor (Qiagen, UK). Frozen PBMCs were thawed at 37 °C and washed in 10 ml of RPMI-1640 containing FBS (10%) prior to resuspension in ATL lysis buffer. DNA quality was confirmed using a bioanalyzer (Agilent, CA, USA). Bisulfate conversion and measurement of DNA methylation were performed by Diagenode SA (Belgium) using the Infinium EPIC 850k Methylation array (Illumina, CA, USA). The data were analyzed using open access R packages. Preprocessing was performed using Minfi^26^, followed by removal of loci associated with SNPs. The calculation of epigenetic age was performed using Hannum^27^, Horvath^17^, PhenoAge^28^, and DunedinPACE^22^ clocks for the PBMC data. The epigenetic age of the muscle tissue was calculated using the Muscle Epigenetic Age Test (MEAT 2.0)^29^. Epigenetic age acceleration or deceleration was defined as the residual value obtained following linear regression of DNA methylation age on chronological age.

### Calculation of senescence gene expression in skeletal muscle tissue

Muscle tissue biopsies (~10 mg) were first homogenized in RLT buffer (Qiagen, Manchester, UK) utilizing a Qiagen Tissue Ruptor (Qiagen, Manchester, UK). RNA was then isolated and treated with DNAse using a commercially available kit following the manufacturer’s protocol (RNeasy Fibrous Tissue Mini Kit, Qiagen Manchester, UK). The quantity and quality of the RNA were measured utilizing a Bioanalyzer (Agilent, CA, USA). Library preparation and RNA‐sequencing were performed by the Genomics Facility at the University of Birmingham using a QuantSeq 3′ kit (Lexogen), and the libraries were sequenced on an Illumina NextSeq 500 platform. The sequence read quality checks were performed using fastQC, and the reads were trimmed using Trimmomatic. Reads were mapped to the hg38 reference human genome using Star Aligner. The enrichment of senescence-associated genes was measured by performing gene set enrichment analysis (GSEA) with the SenMayo gene set^30^.

### T and B cell subset phenotyping

The frozen PBMCs were thawed at 37 °C and washed in 10 ml of RPMI-1640 medium supplemented with 10% FBS prior to resuspension in phosphate-buffered saline (PBS) at 1 × 10^6^ cells/ml. For the identification of T cell subsets, samples were stained for 30 min at 4 °C with combinations of anti-human CD3 (clone REA613, Miltenyi Biotec), anti-human CD4 (clone REA623, Miltenyi Biotec), anti-human CD8 (clone REA734, Miltenyi Biotec), anti-human CCR7 (clone REA546, Miltenyi Biotec), anti-human CD45RA (clone REA562, Miltenyi Biotec), anti-human CD28 (clone REA612, Miltenyi Biotec), anti-human CD57 (clone REA769, Miltenyi Biotec), and anti-human PD1 (CD279) (clone REA1165, Miltenyi Biotec) antibodies. For the identification of B cell subsets, combinations of anti-human CD19 (clone REA675, Miltenyi Biotec), anti-human CD27 (clone REA499, Miltenyi Biotec), anti-human IgD (clone REA740, Miltenyi Biotec), anti-human CD24 (clone REA843, Miltenyi Biotec), and anti-human CD38 (clone REA671, Miltenyi Biotec) antibodies were used. After incubation, the cells were washed twice in PBS and analyzed using a MacsQuant X (MACSQuant® X, Miltenyi Biotec).

Data analysis was performed using FlowJo software (v10.8.1). A total of 20,000 T cells (CD3+) were gated and divided into four subsets based on CD45RA and CCR7 expression as follows: naïve (CD45RA^+^CCR7^+^), central memory (CD45RA^−^CCR7^+^), effector memory (CD45RA^−^ CCR7^−^), and terminally differentiated effector memory (CD45RA^+^CCR7^−^) subsets. Senescent and exhausted T cells were defined as CD3^+^CD28^−^CD57^+^ and CD3^+^PD1^+^ T cells, respectively.

A total of 5,000 B cells (CD19^+^) were divided into naïve (CD27^−^IgD^+^), switched memory (CD27^+^IgD^+^), unswitched memory (CD27^−^IgD^+^), exhausted (CD27^−^IgD^-^), regulatory B (CD24^high^ CD38^high^), naïve (CD24^int^CD38^int^), plasma blast (CD24^low^ CD38^high^), and memory (CD24^high^ CD38) subsets. The gating strategies used for the T and B cell subsets have been previously reported^31^.

### Regulatory T cell phenotyping

PBMCs were resuspended in 50 µl of PBS and stained with anti-human CD3 (Clone REA613, Miltenyi Biotec), CD4 (Clone REA623, Miltenyi Biotec), and CD25 (REA570, Miltenyi Biotec) antibodies for 30 min at 4 °C. After incubation, the cells were washed twice with PBS and fixed with Foxp3 Fix Perm solution (Miltenyi Biotec) for 30 min at room temperature, followed by washing and staining with anti-human Foxp3 (clone REA1253) diluted in permeabilization buffer (Miltenyi Biotec) for 30 min at 4 °C. After incubation, the cells were washed and analyzed using a MACSQuant® X (Miltenyi Biotec). Regulatory T cells were defined as CD3^+^, CD4^+^, CD25^+^, or Foxp3^+^ cells. The gating strategy used has been previously described^31^.

### Calculation of the IMM-AGE score

The IMM-AGE score was calculated using a subset of eight immune cell types (total T cells, naïve CD4 + T cells, CD4+ effector memory T cells, CD8+ effector memory T cells, CD8 + EMRA T cells, CD28 − CD8 + T cells, CD57 + CD8 + T cells, and regulatory T cells) as previously reported^31^.

### Quantification of serum cytokine concentrations

The concentration of serum GDF-15 was measured using a commercially available ELISA kit (DY957, R&D Systems, MN, USA) following the manufacturer’s protocol. Serum levels of TNFα, TNFβ, and tyrosine-kinase receptor encoded by the KIT locus (KITLG) (also known as stem-cell factor (SCF)) were measured as part of a multiplex magnetic bead assay (Bio-Plex Pro Human Cytokine Screening Panel, 48-Plex #12007283, Bio-Rad, Hertfordshire, UK) following the manufacturer’s protocol in combination with a Bioplex 200 (Bio-Rad, Hertfordshire, UK). All the samples were measured in duplicate.

### Statistical analysis

Data analysis was performed using GraphPad Prism v9. The normality of the data was established by performing Shapiro–Wilk analysis. For normally distributed data, statistical significance was determined by performing unpaired *t* tests or ANOVA with Dunnett’s post hoc tests for datasets with multiple groups. The statistical significance of nonparametric data was assessed by performing a Mann‒Whitney U or Kruskal‒Wallis test. Linear regressions were performed to test for associations between measures of epigenetic age or IMM-AGE and other variables. A *p* value of <0.05 was considered to indicate statistical significance.

## Results

### Participant characteristics

There was no significant difference in age between the healthy controls (49.7 ± 15 years, *n* = 18) and CLD patients (55.5 ± 10.0 years, *n* = 38; *p* = 0.18). Additional characteristics, including body composition, skeletal muscle mass, physical function, blood markers, medications, and the clinical profile of CLD patients, are presented in Table 1. Compared with the healthy controls, the CLD group displayed increased adiposity, with a significantly greater dry body mass index (BMI, corrected for ascites and/or peripheral edema; *p* = 0.04), dry body weight (*p* = 0.01), waist-to-hip ratio (*p* < 0.0001), body fat mass (*p* = 0.035), and quadricep intramuscular adipose tissue (*p* < 0.0001). Regarding muscle mass and function, CLD patients exhibited a significantly lower quadricep peak anatomical cross-sectional area (ACSA) (*p* = 0.02) and nondominant peak knee extensor torque (*p* = 0.002), but there was no significant difference in quadricep volume. Moreover, the maximal hand grip strength was lower in the CLD group, but this difference did not reach statistical significance (*p* = 0.052).

**Table 1:** Demographic data for healthy controls and CLD patients.

	Healthy controls	CLD patients	*p* value
Demographics			
* N*	18	38	–
Male/female	11/7	23/15	–
Age (years)	49.7 ± 15.0	55.5 ± 10.0	0.2
Height (m)	1.70 ± 0.1 (*n* = 17)	1.74 ± 0.1	0.4
Current smoker (*n*, %)	1 (6.6) (*n* = *15)*	4 (10.5)	–
Ex-smoker (*n*, %)	2 (13.3) (*n* = *15)*	16 (42.1)	–
Current alcohol consumption (units/week)	2 (5) (*n* = *15)*	0 (0)	**<0.001**
Co-morbidities (*n*, %)			
Cardiovascular disease	0	4 (10.5)	**-**
Chronic kidney disease	0	8 (21.0)	**-**
COPD	0	6 (15.7)	**-**
Diabetes mellitus	0	10 (26.3)	**-**
Hypercholesterolemia	0	6 (15.7)	**-**
Hypertension	0	11 (28.9)	**-**
Insulin-dependent diabetes mellitus	0	8 (21.0)	**-**
Metabolic profile			
BMI (kg/m^2^)	24.6 ± 3.5 (*n* = *17)*	30.3 ± 6.4	**0.001**
Body fat (%)	27.5 ± 7.7 (*n* = 16)	30.3 ± 10.7	0.4
Body fat mass (kg)	19.9 ± 6.4 (*n* = 16)	28.9 ± 14.1	**0.04**
Dry BMI (kg/m^2^)	73.0 ± 12.3	27.9 ± 5.6	**0.04**
Dry weight (kg)	73.0 ± 12.3	86.26 ± 18.7	**0.01**
Fat-free mass (kg)	51.0 ± 11.5 (*n* = 16)	62.7 ± 12.2	**0.002**
HbA1c (mmol/mol)	33.8 ± 4.3	38.3 ± 15.7 (*n* = 35)	0.7
Quad IMAT (%)	5.2 ± 1.8	10.7 ± 3.6	**<0.0001**
Waist: hip	0.8 ± 0.06	1.0 ± 0.08 (*n* = 37)	**<0.0001**
Weight (kg)	73.0 ± 12.3 (*n* = 17)	91.7 ± 21.1	**<0.0001**
Skeletal muscle structure and function			
Maximal dominant hand grip strength (kg)	36.9 ± 10.5	31.6 ± 8.6	0.05
Peak knee extensor toque (Nm)	142.5 ± 51.1	99.2 ± 41.4	**0.002**
Peak quad ACSA (cm^2^)	64.8 ± 17.8	55.1 ± 11.9 (*n* = 34)	**0.02**
Overall daily activity (mg)	29.2 ± 8.9 (*n* = 17)	19.0 ± 7.5 (*n* = 30)	**<0.0001**
Quad volume (cm^3^)	1227.3 ± 393.5	1083.1 ± 259.9 (*n* = 34)	0.1
Specific force (Nm/cm^2^)	2.2 ± 0.5	2.0 ± 0.6 (*n* = 34)	0.19
VL muscle thickness (cm)	2.4 ± 0.5	2.2 ± 0.4	**0.04**
Disease type (*n*, %)			
ArLD	–	17 (45)	–
Immune mediated	–	15 (39)	–
NAFLD	–	6 (16)	–
CLD severity			
Childs-Pugh Score	–	11.08 ± 12.3	–
LFI	2.8 ± 0.6	3.6 ± 0.6	**<0.0001**
MELD	–	12.08 ± 5.1	–
UKELD	–	49.71 ± 10.7	–
Complications of CLD (*n*, %)			
Ascites	–	26 (68.4)	–
Encephalopathy	–	22 (58.0%)	–
Large volume paracentesis	–	7 (18.4%)	–
Portal hypertension	–	33 (86.8)	–
Portal vein thrombosis	–	5 (13.2)	–
Spontaneous bacterial peritonitis	–	9 (23.7)	–
TIPS	–	1 (2.6)	–
Medications			
Beta blockers	0	14 (36.8)	–
Diuretics	0	24 (63.2)	–
Rifaximin	0	22 (57.9)	–
Antibiotic prophylaxis	0	8 (21.0)	–
Blood analysis			
Albumin	42.7 ± 2.6	34.5 ± 5.8	**<0.0001**
ALP	71.5 (18.3)	165.0 (171.0)	**<0.0001**
ALT	18 (6)	30.5 (33.6)	**0.002**
Bilirubin	12.0 (8.0)	32.5 (27.5)	**<0.0001**
Creatinine	81 (20)	72.5 (31)	0.5
INR	1.0 (0.1)	1.2 (0.15)	**<0.0001**
Platelets	237 (61.3)	96 (63.8)	**<0.0001**
Potassium	4.2 ± 0.24	4.3 ± 0.39	0.2
Sodium	139.9 ± 1.8	137.4 ± 3.3	**0.005**
Urea	5.1 (1.2)	5.9 (4.9)	0.2
WCC	4.9 (0.7)	4.2 (2.9)	**0.001**

Parametric data are reported as the mean ± SD, and nonparametric data are reported as the median (IQR). Statistical significance was determined by either unpaired Student’s *t* test or the Mann–Whitney test for parametric and nonparametric data, respectively. Dry body weight/BMI was determined by subtracting the percentage of fluid based on clinical examination for mild (5%), moderate (10%), and severe (15%) ascites and/or mild (5%) and moderate (10%) peripheral edema. The quadricep muscle mass was measured relative to the dominant leg, and the maximal knee extensor force was measured in the nondominant leg.

*ACSA* anatomical cross-sectional area, *ALP* alkaline phosphatase, *ALT* alanine aminotransferase, *COPD* chronic obstructive pulmonary disease; *INR* international normalized ratio, *LFI* liver frailty index, *MELD* model for end-stage liver disease, *TIPS* transjugular intrahepatic portosystemic shunt, *UKELD* UK model for end-stage liver disease, *WCC* white cell count, *VL* vastus lateralis.

Bold values were utilised to emphasize significant values.

### CLD is associated with increased skeletal muscle epigenetic aging

**Fig. 1:**
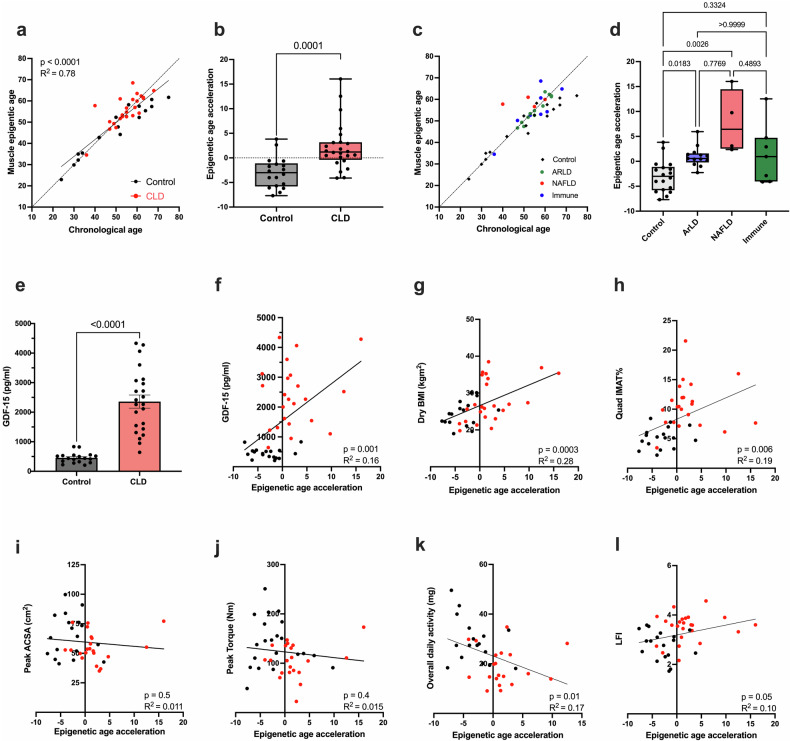
Evidence of accelerated skeletal muscle aging in patients with chronic liver disease. **a** Scatter plot comparing skeletal muscle epigenetic age with chronological age for patients with CLD (*n* = 24) and healthy control individuals (*n* = 18). **b** Skeletal muscle epigenetic age acceleration in CLD patients and healthy controls. **c** Scatter plot comparing skeletal muscle epigenetic age with chronological age for CLD etiology subgroups and healthy controls (ArLD, *n* = 13; NAFLD, *n* = 4; immune-mediated, *n* = 7). **d** Skeletal muscle epigenetic age acceleration for CLD etiology subgroups and healthy controls. **e** Serum concentration of GDF-15 in matched muscle biopsy samples from CLD patients (*n* = 24) and healthy controls (*n* = 18). **f** Scatter plot displaying the association between serum GDF-15 concentrations and skeletal muscle epigenetic age acceleration in CLD patients and healthy controls. **g**–**l** Scatter plots showing the associations of muscle epigenetic age with BMI, quadricep intramuscular adipose tissue, quad peak ACSA, peak isokinetic knee extensor torque, overall daily activity, and liver frailty index for CLD patients and healthy controls. The black symbols denote healthy controls, and the red symbols denote CLD patients. Normally distributed data are presented as the mean ± SEM. Nonparametric data are presented as box and whisker plots with medians and lower and upper quartiles. IMAT intermuscular adipose tissue, ACSA anatomical cross-sectional area, ArLD alcohol-related liver disease, LFI liver frailty index, NAFLD nonalcoholic fatty liver disease.

To investigate whether accelerated epigenetic aging is associated with the development of sarcopenia in CLD patients, the epigenetic age of skeletal muscle tissue obtained from CLD patients and matched healthy controls was determined using the muscle-specific epigenetic clock described by Viosin et al.^29^. Overall, this model was robust, with epigenetic age demonstrating a highly significant (*p* < 0.0001, *R*^2^ = 0.78) positive correlation with chronological age (Fig. 1a). Epigenetic age acceleration was significantly greater in the CLD cohort (2.2 ± 4.8) than in the matched healthy controls (−3.0 ± 3.2; *p* = 0.0001, Fig. 1b). Healthy controls predominantly displayed a younger epigenetic age compared to their chronological age (up to −13.3 years), particularly in older individuals (Fig. 1a,b). In contrast, the epigenetic age of the CLD cohort was greater than their chronological age (up to +17.8 years) (Fig. 1a, b). To examine whether there are differences in epigenetic age acceleration depending upon CLD etiology (Fig. 1c), comparisons were made among individuals with nonalcoholic fatty liver disease (NAFLD), alcohol-related liver disease (ArLD), and immune-mediated liver disease, specifically primary sclerosing cholangitis and primary biliary cholangitis (PSC/PBC). Despite the limited number of participants, age was significantly greater in both the ArLD (*p* = 0.01) and NAFLD (*p* = 0.0026) subgroups than in the healthy controls (Fig.1d). No significant difference in age acceleration was observed between the immune-mediated subgroup and healthy controls (*p* = 0.3), but this disease subgroup exhibited the greatest intragroup variability. No significant differences in age acceleration were identified between disease etiologies (Fig. 1d).

To further investigate an accelerated aging phenotype in the skeletal muscle of CLD patients, the association of epigenetic muscle aging with circulating levels of the GDF-15 cytokine, a well-characterized biomarker of aging^32^, was determined. GDF-15 concentrations were significantly higher in the CLD group (5.2-fold, *p* < 0.001, Fig. 1e) and demonstrated a positive correlation with muscle epigenetic age acceleration (*p* < 0.001, *R*^2^ = 0.16, Fig. 1f). Because there was evidence of accelerated skeletal muscle aging in patients with CLD, the present study investigated how this process is related to muscle physiology and physical function. There was a significant positive correlation between muscle epigenetic age acceleration and both dry BMI (*p* = 0.0003, *R*^2^ = 0.28, Fig. 1g) and quadricep IMAT (*p* = 0.006, *R*^2^ = 0.19, Fig. 1h). Despite the greater adiposity in CLD patients than in the healthy controls (Table 1), muscle epigenetic age acceleration was significantly greater in CLD patients when comparing only lean individuals (dry BMI < 25, *p* = 0.0391), and the quad IMAT also demonstrated a positive correlation with muscle epigenetic age acceleration (*p* = 0.017, *R*^2^ = 0.28, Supplementary Fig. 1a, b). Additionally, muscle epigenetic age did not differ between diabetic and nondiabetic individuals, and it was not associated with HbA1c (Supplementary Fig. 1c, d).

No associations were detected between muscle age and either the quadricep peak ACSA (*p* = 0.5) or quadricep strength (*p* = 0.4, Fig. 1i, j). However, muscle age acceleration was associated with activity level (*p* = 0.01, *R*^2^ = 0.17) and the liver frailty index (LFI), a performance-based test consisting of measures of grip strength, chair stand time, and balance (*p* = 0.05 *R*^2^ = 0.10, Fig. 1k, l). As systemic inflammation may be a driver of muscle age acceleration, the present study examined this relationship. There was a significant positive correlation between muscle age and the circulating neutrophil count (*p* = 0.014, *R*^2^ = 0.14, Supplementary Fig. 1e); however, no associations between white cell count (WCC) and C-reactive protein (CRP) were detected (Supplementary Fig. 1f, g). When considering additional clinical factors that may impact muscle epigenetic aging, no associations of muscle aging with liver disease severity (MELD or UKELD scores), history of spontaneous bacterial peritonitis infection, or use of diuretics were detected (Supplementary Fig. 1h-k).

### Enrichment of senescence-associated genes in the skeletal muscle of CLD patients

**Fig. 2:**
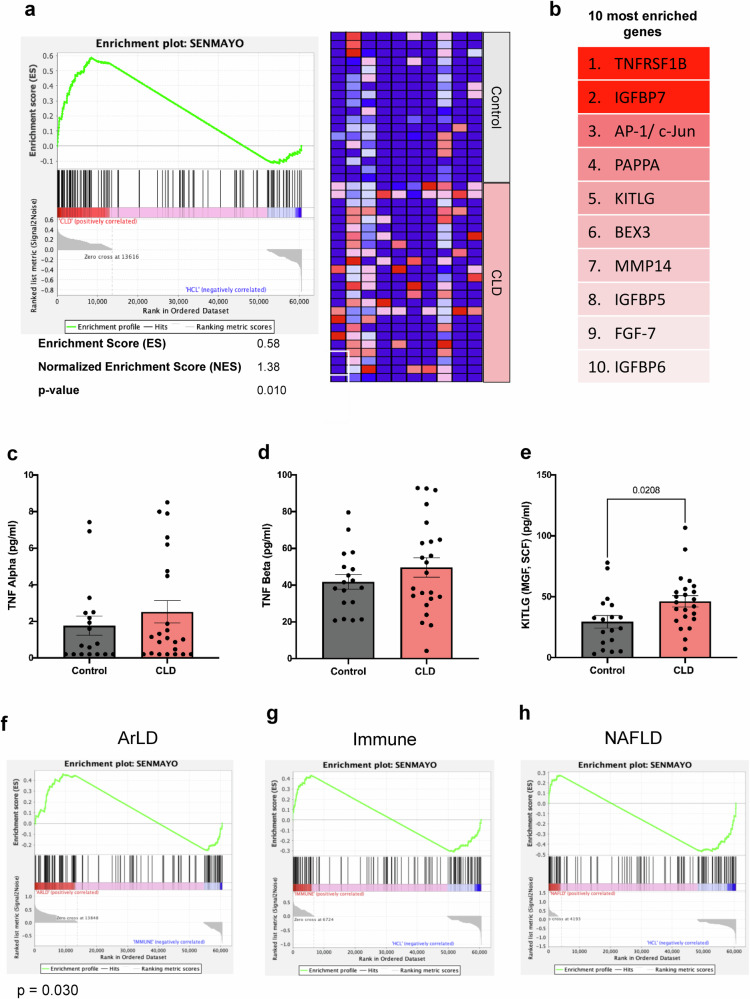
Evidence of increased senescence in patients with chronic liver disease. **a** SenMayo gene set enrichment analysis of the skeletal muscle tissue of CLD patients (*n* = 24) compared to that of healthy controls (*n* = 18). The data are presented as an enrichment plot (left) and a heatmap of the top 10 most enriched SenMayo genes in the CLD group (right). **b** Top 10 most enriched SenMayo genes in the skeletal muscle tissue of CLD patients **c**, **d** Serum concentrations of TNF-alpha and TNF-beta in CLD patients (*n* = 23) and healthy controls (*n* = 18). **e** Serum concentrations of KITLG (pseudonyms: stem cell factor (SCF) and mast cell growth factor (MGF)) in patients with CLD and healthy controls. **f**–**h** Enrichment plots following gene set enrichment analysis of the SenMayo gene set in the skeletal muscle tissue of the ArLD (*n* = 13), immune-mediated (*n* = 7), and NAFLD (*n* = 4) subgroups in comparison to healthy controls. The data are presented as the mean ± SEM. ArLD alcohol-related liver disease, NAFLD nonalcoholic fatty liver disease.

As increased cellular senescence is a well-defined hallmark of aging, the recently published SenMayo gene set^30^ was utilized to estimate the senescent cell burden in skeletal muscle tissue. In line with greater epigenetic age acceleration, gene set enrichment analysis revealed a significant (*p* = 0.01) enrichment of the SenMayo gene set in the skeletal muscle tissue of CLD patients compared to healthy control muscle tissue (Fig. 2a). To further validate the functional significance of these findings, the circulating levels of TNFα, TNFβ (the ligand for the most highly enriched gene, TNFRSF1B) (Fig. 2b), and KITLG (an enriched cytokine and a potentially novel biomarker for muscle aging) (Fig. 2b) were measured. The circulating levels of TNFα and TNFβ were not significantly different between the CLD and control groups (Fig. 2c, d), and they did not correlate with muscle epigenetic age (Supplementary Fig. 2a, b). Compared to the healthy controls, KITLG was significantly (*p* = 0.02) elevated in CLD patients by ~50% (Fig. 2e), but circulating KITLG levels were not associated with muscle epigenetic age acceleration (Supplementary Fig. 2c). Upon categorizing the CLD cohort by disease etiology, a significant (*p* = 0.030) enrichment of SenMayo was only found in the ArLD subgroup (Fig. 2f–h).

### Evidence of epigenetic aging in blood cells from patients with CLD

**Fig. 3:**
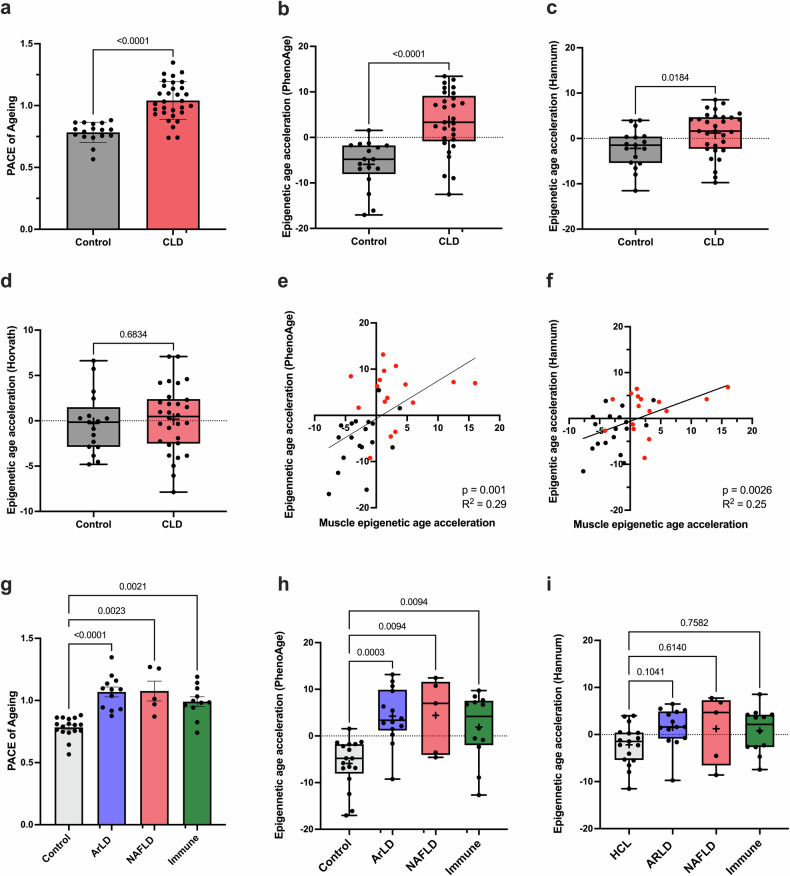
Evidence of accelerated epigenetic aging of the immune system in patients with chronic liver disease. **a** DunedinPACE scores based on the DNA methylome of PBMCs from CLD patients (*n* = 31) and healthy controls (*n* = 17). **b**–**d** Epigenetic age acceleration of PBMCs calculated by the PhenoAge, Horvath, and Hannum clock scores. **e**, **f** Scatter plot showing the relationship between skeletal muscle epigenetic age acceleration and PBMC PhenoAge and Hannum epigenetic age acceleration in patient-matched samples (*n* = 17 CLDs and *n* = 17 controls). **g**–**i** PBMC DunedinPACE scores, PhenoAge epigenetic age acceleration, and Hannum epigenetic age acceleration for CLD patients subgrouped by disease etiology (ArLD, *n* = 14; NAFLD *n* = 5; immune-mediated, *n* = 12) and healthy controls (*n* = 17). The black symbols denote healthy controls, and the red symbols denote CLD patients. The data are presented as the means ± SEMs or as box-and-whisker plots displaying the medians, lower quartiles, upper quartiles, and means (+). ArLD alcohol-related liver disease, NAFLD nonalcoholic fatty liver disease.

Following the observation of accelerated aging and increased skeletal muscle expression of senescence-associated genes in CLD patients, the present study determined whether this accelerated aging phenotype is observed across other relevant systems. To this end, the epigenetic age of PBMCs in the CLD and control groups was determined using multiple epigenetic clocks. These clocks have been shown to be indicators of overall biological aging^17,22^. Analysis of epigenetic aging in PBMCs via the DunedinPACE clock revealed a significant increase (33%) in the aging trajectory of the CLD group (1.04 ± 0.027) compared to that of the control group (0.78 ± 0.020; *p* < 0.0001, Fig. 3a). Similarly, the increase in the epigenetic age of PBMCs in the CLD group was also significantly greater than that in the control group, as calculated using either the PhenoAge (*p* < 0.0001) or Hannum (*p* = 0.01) clock (Fig. 3b, c). Epigenetic age acceleration calculated by the Horvath clock did not significantly differ between the groups (Fig. 3d). Additionally, there was a positive correlation between matched muscle tissue and PBMC epigenetic age acceleration, as calculated by both the PhenoAge clock (*p* = 0.001, *R*^2^ = 0.29, Fig. 3e) and the Hannum clock (*p* = 0.003 *R*^2^ = 0.25, Fig. 3f), demonstrating a consistent aging phenotype across muscle tissue and PBMCs. Despite the small sample size, analysis of the CLD cohort by disease etiology indicated that the DunedinPACE scores and PhenoAge acceleration were significantly greater for all etiologies than for the control group, with limited variation between groups (Fig. 3g, h). However, no significant difference between the healthy controls and CLD patient subgroups was detected using the Hannum clock (Fig. 3i).

**Fig. 4:**
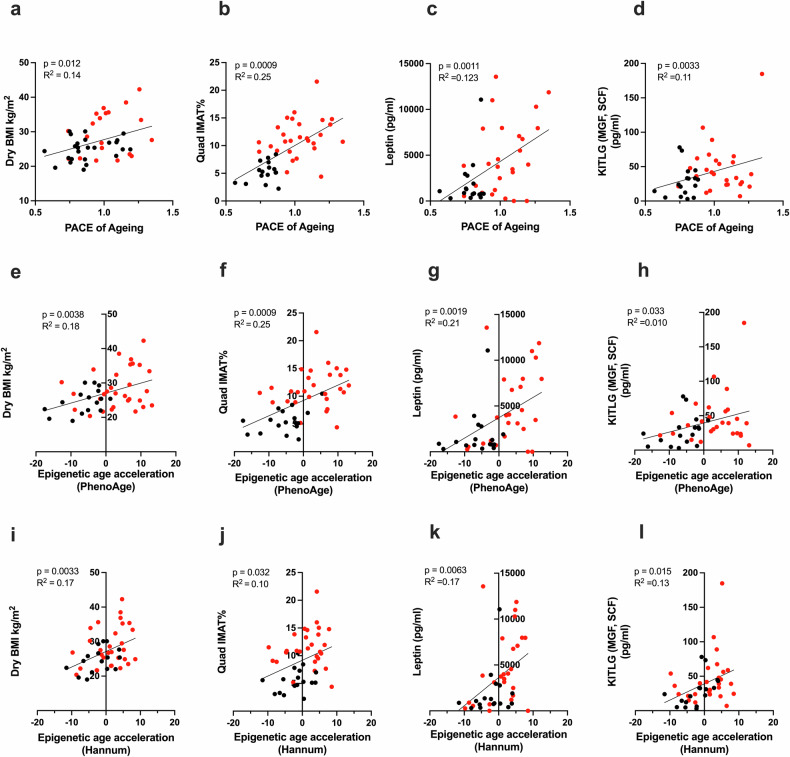
Associations of PBMC age with physical parameters. **a**–**d** Scatter plots showing the relationships of PBMC epigenetic DunedinPACE score with BMI, quad IMAT, serum leptin concentrations, and serum KITLG concentrations. **e**–**h** Scatter plots showing the relationships of PhenoAge epigenetic age acceleration with BMI, quad IMAT, serum leptin concentrations, and serum KITLG concentrations**. i**–**l** Scatter plots showing the relationships of Hannum epigenetic age acceleration with dry BMI (CLD, *n* = 30; controls, *n* = 16), quad IMAT (CLD, *n* = 29; controls, *n* = 16), serum leptin concentrations (CLD, *n* = 26; controls, *n* = 17), and serum KITLG concentrations (CLD, *n* = 27; controls, *n* = 17). The black symbols denote healthy controls, and the red symbols denote CLD patients. ArLD alcohol-related liver disease, IMAT intramuscular adipose tissue, NAFLD nonalcoholic fatty liver disease.

As in the skeletal muscle tissue, PBMC epigenetic age was also associated with increased adiposity, with significant correlations observed between the DundeinPACE score and both dry BMI (*p* = 0.0003, Fig. 4a) and quadricep IMAT (*p* = 0.0009, Fig. 4b). In addition, there was a significant correlation between the DunedinPACE score and the serum levels of leptin (*p* = 0.0011, Fig. 4c), an adipokine associated with adipose tissue burden^32^. Furthermore, there was a significant positive correlation between the DunedinPACE score and serum KITLG (*p* = 0.0033, Fig. 4d), which was identified as a senescence-associated gene enriched in CLD muscle (Fig. 2). In line with this, both PhenoAge and Hannum age acceleration also demonstrated a significant positive association with these parameters (Fig. 4e–l). Further, significantly greater epigenetic aging and a positive correlation between IMAT% and both the DunedinPACE score and PhenoAge acceleration were detected in lean individuals only (dry BMI ≤ 25) (Supplementary Fig. 3a–f), and this effect was independent of diabetes status (Supplementary Fig. 3g–l).

As observed in skeletal muscle, the DunedinPACE scores, PhenoAge acceleration, and Hannum age acceleration were not associated with measures of muscle mass or strength (Supplementary Fig. 4). Similarly, the associations of the epigenetic age of PBMCs with blood markers (CRP, WCC, and neutrophil counts) were limited (Supplementary Fig. 5a–i), with only CRP positively correlating with the DunedinPACE score (Supplementary Fig. 5a). Clinically, the DunedinPACE score (but not PhenoAge or Hannum age acceleration) was also positively associated with the UKELD score (Supplementary Fig. 6a–c), but PBMC epigenetic aging was not associated with the MELD score, diuretic use, or history of spontaneous bacterial peritonitis infection (Supplementary Fig. 6d–l).

### Immune senescence is greater in patients with CLD than in healthy controls

To determine whether accelerated epigenetic aging based on PBMCs and skeletal muscle epigenomes is consistent with an enhanced aging phenotype of the immune system, comprehensive immune cell phenotyping of PBMCs from CLD patients and controls was performed (summarized in Table 2). Clear differences in the immune cell populations of both T and B cells were observed between the CLD and control groups (Table 2). Consistent with greater SenMayo enrichment in skeletal muscle (Fig. 2), the CLD cohort had significantly greater proportions of senescent CD8^+^ T cells (*p* = 0.013), exhausted CD4^+^ T cells (*p* = 0.0003), and regulatory T cells (*p* = 0.0001), which are all markers of an aged immune system. Additionally, lower frequencies of naïve CD4^+^ T cells were observed in the CLD cohort, but this difference did not reach significance (*p* = 0.065, Table 2). Among B cells, there was a higher frequency of exhausted subsets (IGD- CD27^-^, *p* = 0.030) and plasma blasts (CD24^low^ CD38^high^, *p* < 0.0001) but lower proportions of classical switched memory B cells (IGD^-^CD27^+^, *p* = 0.0055) and memory B cells (CD24^high^ CD38^-^, *p* < 0.0001) in the CLD cohort (Table 2).

**Table 2:** Summary of immune cell phenotyping.

	Heathy controls			CLD patients			
Cell population (%)	Median	Mean	SD	Median	Mean	SD	*p* value
T cell subsets							
CD4^+^ CD45 RA^-^ CCR7^-^ (effector memory)	19.1	19.5	7.1	26.0	29.7	16.0	**0.015**
**CD4**^**+**^ **CD45 RA** ^**+**^ **CCR7**^**-**^ **(EMRA)**	7.5	8.3	4.2	10.2	10.2	5.1	0.17
CD4^+^ CD45 RA- CCR7^+^ (central memory)	14.1	15.4	8.4	9.6	11.7	7.8	0.13
CD4^+^ CD45 RA^+^ CCR7^+^ (naïve)	57.0	57.3	11.8	52.0	49.4	17.2	**0.065**
CD8^+^ CD45 RA^-^ CCR7^-^ (effector memory)	19.8	19.0	10.5	23.3	22.2	13.0	0.40
**CD8**^**+**^ **CD45 RA**^**+**^ **CCR7**^**-**^ **(EMRA)**	19.0	20.8	9.7	26.8	28.4	12.8	**0.036**
CD8^+^ CD45 RA^-^ CCR7^+^ (central memory)	2.2	2.6	1.3	4.2	6.0	4.4	**0.0065**
CD8^+^ CD45 RA^+^ CCR7^+^ (naïve)	49.0	51.0	13.3	45.0	45.2	16.0	0.19
(FOXp3^+^) (Regulatory T cells)	2.2	2.6	1.3	4.2	6.0	4.4	**0.0001**
Senescent T cells							
CD4^+^ CD28^-^ CD57^+^	1.7	2.4	2.8	2.4	4.4	5.0	0.40
CD8^+^ CD28^-^ CD57^+^	5.3	4.9	3.0	6.7	13.4	14.7	**0.013**
Exhausted T cells							
CD4^+^ PD1^+^	6.9	9.1	4.9	16.4	18.2	9.1	**0.0003**
CD8^+^ PD1^+^	13.4	14.5	10.2	9.0	10.7	7.4	0.25
B cell subsets							
IGD^-^ CD27^-^ (exhausted)	8.8	9.9	5.5	13.7	19.5	15.5	**0.030**
IGD^-^ CD27^+^ (classical switched memory)	23.7	24.3	11.4	13.1	14.8	10.4	**0.0055**
IGD^+^ CD27^-^ (Naïve)	51.4	16.0	3.8	55.9	52.8	23.7	0.84
IGD^+^ CD27^+^ (nonswitched)	12.0	14.4	8.4	9.7	12.8	14.1	0.15
CD24^int^ CD38^int^ (naïve B cells)	51.2	51.3	17.0	59.3	55.5	17.7	0.41
CD24^low^ CD38^high^ (plasma blasts)	0.8	1.4	1.9	8.2	12.2	12.6	**<0.0001**
CD24^high^ CD38- (memory B cells)	22.2	21.5	9.4	6.0	7.7	5.7	**<0.0001**
CD24^high^ CD38^high^ (regulatory B cells)	4.6	4.4	1.9	4.4	5.5	3.6	0.56

Statistical significance was determined by either unpaired Student’s *t* test or the Mann–Whitney test for parametric and nonparametric data, respectively.

Bold values were utilised to emphasize significant values.

**Fig. 5:**
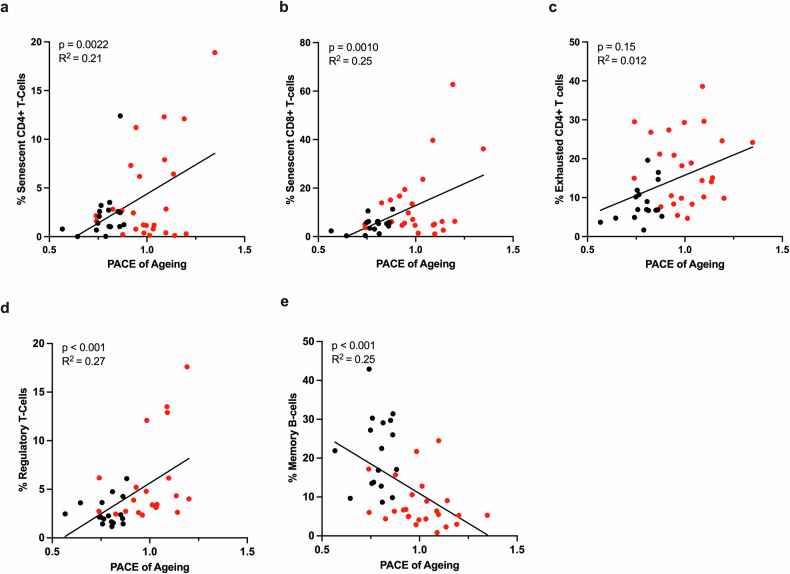
Association of PBMC DunedinPACE scores with immune cell subsets. Scatter plots displaying the association of PBMC epigenetic DunedinPACE score and **a** percent of senescent CD4^+^ T cells (CLD *n* = 24, control *n* = 17), **b** percent of senescent CD8^+^ T cells (CLD, *n* = 25; control, *n* = 17), **c** percent of exhausted CD4 + T cells (CLD *n* = 25, control *n* = 17), **d** percent of regulatory T cells, and **e** percent of memory B cells (CLD *n* = 25, control *n* = 17). The black symbols denote healthy controls, and the red symbols denote CLD patients.

The present study also explored whether increases in such age-associated immune cell populations are associated with epigenetic aging. There were significant positive correlations of the DunedinPACE score with senescent CD4^+^ T cells, exhausted CD4^+^ T cells, senescent CD8^+^ T cells, and regulatory T cells (Fig. 5a–d). In addition, there was a negative correlation of the DunedinPACE score with memory B cells (*p* < 0.001, *R*^2^ = 0.25, Fig. 5e). Similar associations were also observed for PhenoAge and Hannum epigenetic age acceleration (Supplementary Fig. 7).

**Fig. 6:**
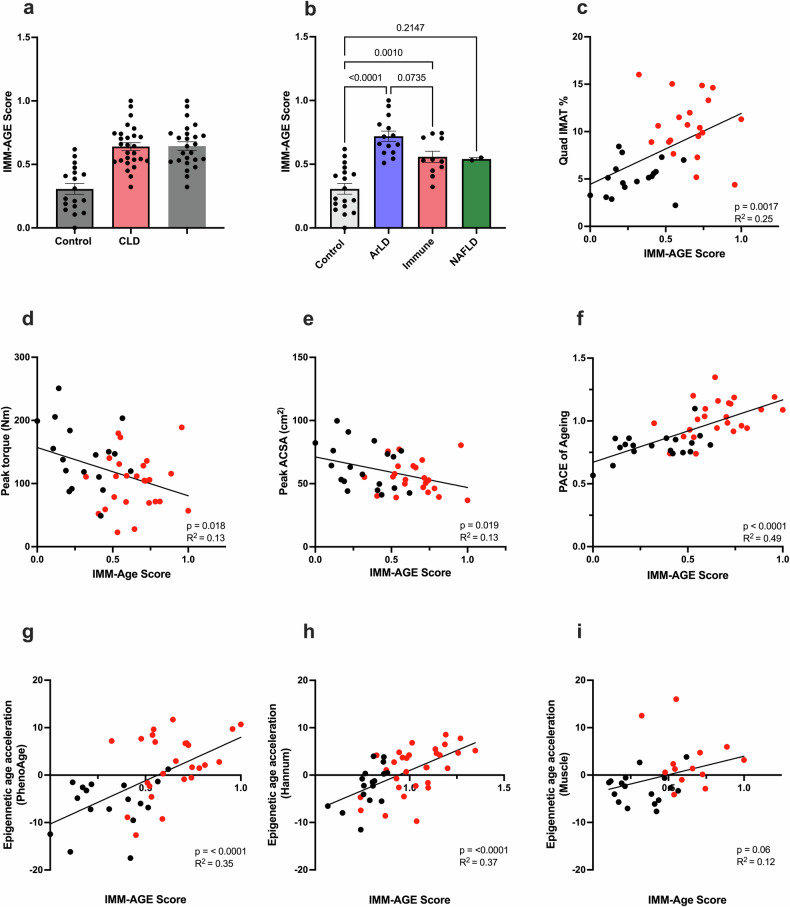
Patients with chronic liver disease exhibit accelerated immune system aging according to phenotypic measures. **a** IMM-AGE scores calculated for immune cell subsets in CLD patients (*n* = 27) and healthy controls (*n* = 18). **b** IMM-AGE scores calculated from immune cell subsets for CLD patients subgrouped by disease etiology (ArLD, *n* = 14; NAFLD, *n* = 2; immune-mediated, *n* = 11) and healthy controls (*n* = 18). **c**–**e** Scatter plots showing the associations between the IMM-AGE scores of the PBMCs and the physical parameters of quad IMAT%, peak knee extensor torque, and peak quadricep ACSA (*n* = 26 CLD, *n* = 17 control). **f**–**h** Scatter plots showing the association of PBMC IMM-AGE scores with PBMC epigenetic DunedinPACE scores, PhenoAge epigenetic age acceleration, and Hannum epigenetic age acceleration (CLD, *n* = 25; Control, *n* = 17). **i** Scatter plots showing the association between IMM-AGE scores of PBMCs and age-matched muscle epigenetic acceleration (CLD, *n* = 13; control, *n* = 17). The black symbols denote healthy controls, and the red symbols denote CLD patients. The data are presented as the mean ± SEM. ACSA anatomical cross-sectional area, ArLD alcohol-related liver disease, IMAT intramuscular adipose tissue, NAFLD nonalcoholic fatty liver disease.

The immune cell phenotyping data of CLD patient and control PBMCs were used to calculate the IMM-AGE score for overall immune aging using a modified metric of the original algorithm^31^. In line with the epigenetic data, the IMM-AGE scores of the CLD cohort were 2-fold greater than those of the healthy control cohort (*p* < 0.0001; Fig. 6a). Despite the limited sample size, a significantly greater IMM-AGE score was also observed for the ArLD and immune-mediated disease etiologies (Fig. 6b). No significant difference in the IMM-Age score was observed in the NAFLD subgroup; however, sample availability limited this cohort to *n* = 2 (Fig. 6b). Consistent with muscle and PBMC epigenetic aging, the IMM-AGE score of CLD patients was significantly greater than that of lean individuals (*p* = 0.0012, Supplementary Fig. 8d) and correlated with the intramuscular quadricep adipose tissue percentage (*p* = 0.0017, Fig. 6c). In contrast to muscle and PBMC epigenetic aging measures, negative correlations between the IMM-AGE score and both peak knee extensor torque (*p* = 0.018, *R*^2^ = 0.13, Fig. 6d) and quadricep peak ACSA (*p* = 0.019, *R*^2^ = 0.13, Fig. 6e) were observed. The IMM-Age score was also significantly associated with CRP (*p* = 0.0058) but not with WCC or neutrophil count (Supplementary Fig. 8a–c). Clinically, there were no associations of the IMM-age score with diabetes status, liver disease severity, diuretic use, or history of previous spontaneous bacterial peritonitis infection (Supplementary Fig. 8e–j).

Direct comparison of IMM-AGE scores with matched epigenetic age acceleration measures indicated positive correlations of the IMM-AGE scores with the DunedinPACE score (*p* < 0.001, *R*^2^ = 0.49), PhenoAge acceleration (*p* < 0.00041, *R*^2^ = 0.35), and Hannum age acceleration (*p* < 0.0001, *R*^2^ = 0.37), suggesting consistency of age acceleration among various models of biological aging (Fig. 6f–h). However, the association between IMM-Age and muscle epigenetic age acceleration was not statistically significant (*p* = 0.06, *R*^2^ = 0.12, Fig. 6i).

## Discussion

CLD is a debilitating proinflammatory ‘scarring’ condition that often results in the development of age-associated comorbidities (especially physical frailty), leading to reduced quality of life and ultimately increased mortality. Increased systemic inflammation is recognized as a key driver of the aging phenotype^6^, which increases the risk of multiple life-limiting diseases. The present study investigated whether CLD increases the rate of biological aging in skeletal muscle and in the immune system. These biological systems with known hallmark mechanisms of aging were also investigated to help explain the increased incidence of sarcopenia^23^ and reduced immunity^33^ in this patient population. The present findings provide the first evidence of increased biological aging in patients with CLD across these two biological systems utilizing epigenetic and immune phenotype-based measures. Clinically, the identification of a divergence of biological age from chronological age, or the presence of a negative aging trajectory, may highlight CLD patients at greatest risk of disease progression, allowing early therapeutic intervention, including medicines that directly modulate aging processes^34^.

It has previously been reported that patients with CLD display hallmarks of aging, including reduced telomere length in liver tissue, hepatocytes, and leukocytes, and this telomere attrition is positively associated with mortality risk and hepatic fibrosis^35^. In line with this, the present study identified greater epigenetic age acceleration in the skeletal muscle tissue of CLD patients, suggesting that epigenetic muscle aging may be a contributing factor to the development of muscle dysfunction, which has been reported in up to 70% of patients with CLD^36^. Aging is also associated with a chronic increase in circulating proinflammatory cytokines and a decrease in the level of anti-inflammatory cytokines, a process referred to as ‘inflammageing’^37^. GDF-15 has recently gained recognition as a key age-associated cytokine, with elevated serum concentrations positively correlated with all-cause mortality^38^, multimorbidity^39^, frailty^40^, and sarcopenia^41^. Moreover, Kim-Muller et al. demonstrated that GDF-15 neutralization restores both muscle mass and function in mice^42^. In line with these data and consistent with other reports on CLD^43^, the present study observed significantly higher circulating levels of GDF-15 in CLD patients, which were positively associated with muscle epigenetic age acceleration, further supporting an aged phenotype of skeletal muscle in CLD patients.

Although the mechanisms that drive age-related epigenetic changes are not fully understood, elevated levels of circulating factors, including proinflammatory cytokines, such as TNFα^44^, IL-6^45^, and IL-12^46^, may play a role in modulating epigenetic modifications of DNA. Similarly, increased adiposity, which is also strongly associated with chronic low-grade inflammation^47^, has been reported to drive epigenetic age acceleration in other tissues, including the liver^48^. Therefore, it is possible that alterations in circulating factors, such as increased levels of proinflammatory cytokines or ammonia following primary liver dysfunction, may drive epigenetic changes in secondary tissues, such as skeletal muscle, negatively impacting their aging trajectory. However, it will be important to elucidate key factors that drive epigenetic aging and those that become elevated secondary to age-associated changes in cellular function.

In addition to modifications of the muscle epigenome, it is also possible that the epigenetic muscle aging observed in the present study may be attributed to differential epigenome content in CLD patients, reflecting changes in muscle tissue composition. We have recently reported evidence of significantly greater intramuscular adipose tissue infiltration within the quadricep muscle of CLD patients^23^. In addition, a shift in fiber-type composition, such as a loss of type II fibers typically observed with age, may also have impacted the epigenetic content of the CLD cohort in the present study^49^.

Along with greater muscle epigenetic age, the skeletal muscle tissue of CLD patients was enriched for the novel SenMayo gene set^30^, suggesting a greater presence of senescent cells within the muscle tissue of CLD patients, another hallmark of aging. Despite the terminally differentiated nature of muscle fibers, increased senescent cell burden has recently been reported in the skeletal muscle of older individuals, which may be driven by a p21-mediated mechanism^50,51^. Importantly, increased muscle senescence has been shown to negatively correlate with markers of muscle function, as well as impair muscle regeneration and promote fibrosis in mice^50,52^. Conversely, targeted removal of senescent cells with senolytic compounds improves grip strength and increases muscle satellite cell proliferation in mice^50,52^. Therefore, senolytic-based therapeutic interventions may provide a novel and feasible strategy to maintain or improve muscle function in patients with CLD. Similar approaches have recently been shown to improve physical function in other chronic inflammatory disease states, specifically idiopathic pulmonary fibrosis^14^.

Utilizing the IMM-AGE score^53^, the present study also demonstrated a significant increase in indicators of aging in the immune cells of patients with CLD, agreeing with previous data demonstrating immune cell telomere attrition in CLD patients^54^. Unlike epigenetic age in either muscle tissue or PBMCs, the IMM-AGE score correlated with measures of skeletal muscle mass and may therefore be a more clinically relevant model for predicting decreases in skeletal muscle mass and function. Additionally, it is well established that immune cells infiltrate skeletal muscle and contribute to the regulation of muscle biology^55^. Therefore, increased numbers of senescent immune cells may have contributed to the increased muscle SenMayo score and the localized inflammation within the muscle itself, thus impairing function. The positive association of neutrophil counts with muscle age acceleration lends support to this hypothesis, further indicating a role for immune-muscle cellular crosstalk as a possible driver of muscle epigenetic aging. In addition, immune system aging may also play an important role in driving IMAT accumulation, as a positive association between immune system aging and IMAT was still observed when only considering lean individuals. Collectively, these findings may help to explain the high prevalence of sarcopenia in CLD patients.

In addition to the increased IMM-AGE score, there was greater epigenetic aging of PBMCs in CLD patients. Epigenetic age acceleration was evident with the DunedinPACE score, which assesses the pace of ageing, indicating that the aging trajectory, rather than chronological age prediction, may more accurately reflect disease progression. In line with this, the DunedinPACE epigenetic clock is the only clock that is positively associated with liver disease severity^22^. Notably, the present study reported less age acceleration with first-generation clocks (Horvath and Hannum), potentially reflecting less consideration of phenotypic and health-related data during the development of these clocks in comparison to the second- and third-generation clocks, such as the DunedinPACE clock. Additionally, PBMC epigenetic aging was positively correlated with the IMM-AGE score, which demonstrated consistent biological age acceleration with both methods. Therefore, PMBC epigenetic clocks may provide useful clinical biomarker tools to identify CLD patients at the greatest risk of mortality.

The present study also identified a significant positive correlation between epigenetic aging of the immune system and skeletal muscle tissue epigenetic age acceleration, indicating that CLD is associated with epigenetic aging across different biological systems. Similarly, Sillanpää et al. reported a correlation of epigenetic age across muscle tissue and blood samples obtained from matched individuals^56^. Translationally, PBMC epigenetic clocks have a greater capability for high-throughput analysis for measures of muscle aging compared to the immune cell phenotyping necessary for IMM-AGE calculation or obtaining muscle biopsy samples.

Although all CLD patients had advanced liver disease, the present cohort comprised individuals with ArLD, NAFLD, or immune-mediated disease etiologies. Because there is evidence that liver dysfunction is also driven by etiology-specific mechanisms, the present study evaluated whether disease etiology may differentially impact biological aging. Significant differences were not detected in the epigenetic aging of the immune system or skeletal muscle among the CLD subgroups, which may have been due to insufficient power attributable to the limited number of patients, particularly within the NAFLD and immune-mediated disease subgroups. Despite being associated with the most severe disease phenotype, the ArLD group displayed modest increases in age acceleration scores with limited variation, particularly in muscle, whereas the NAFLD and immune-mediated subgroups included individuals with the greatest muscle age acceleration and more intragroup variability.

A major limitation of the present study was the small sample size, which limited the ability to determine the role of disease etiology in terms of epigenetic and immune age. Therefore, it will be important to confirm the present findings in a larger cohort and with studies specifically designed to examine etiology-associated differences in biological aging. Although the controls were age- and sex-matched, some characteristics differed between the groups, such as BMI, which may have partially contributed to some of the differences between the control and CLD groups. However, the deep phenotyping of the CLD cohort is a compensating strength; this is one of the first studies to obtain skeletal muscle biopsies from patients with CLD and to investigate the epigenome to determine its impact on biological aging trajectories. Additionally, the present study focused only on skeletal muscle tissue and the immune system, although CLD can be associated with the development of other age-related comorbidities, such as osteoporosis^57^. Although challenging due to tissue availability, it would be valuable to determine whether age acceleration may similarly play a role in the development of other comorbidities. For the immune data, the immune compromise suggestion needs to be confirmed by determining vaccine efficacy and its association with the IMM-AGE data in CLD patients. As reduced vaccine efficacy is observed in patients with CLD^58^, the IMM-AGE score may identify patients likely to display reduced vaccine efficacy and need booster vaccinations. Finally, the present study only performed DNA methylation analysis of bulk muscle tissue and PBMCs. The increasing capability to perform both single-cell and single-nuclear DNA/RNA analysis may help identify key cell types at the greatest risk of age acceleration, thereby allowing more specific therapeutic targets to be defined. In the context of CLD, a critical next step will be to understand whether treatment interventions for CLD, such as curative liver transplantation or pharmacological intervention, reverse epigenetic or immune aging and improve the aging trajectory of CLD patients. Such reversal of epigenetic age has been demonstrated in studies utilizing exercise, diet, and pharmacological interventions in both mice and humans^59,60^. In the wider context, it will be important to expand the present findings into other disease cohorts that display clear links to sarcopenia, such as chronic kidney disease or rheumatoid arthritis, to determine whether accelerated muscle or immune aging is also present in these individuals. Additionally, it will be important to further consider the impact of medications used to treat chronic inflammatory conditions on biological aging and sarcopenia, with emerging data suggesting that medications, such as diuretics, may be associated with sarcopenia development^61^.

In conclusion, patients with CLD often present with significant frailty, sarcopenia, and impaired immune function. However, the mechanisms driving the development of such typically age-related comorbidities are not fully understood. The present study demonstrated that CLD is associated with epigenetic and transcriptomic reprogramming in both skeletal muscle and the immune system, driving an accelerated aging phenotype. Such inherent cellular aging may contribute to the development of sarcopenia and immune dysfunction in patients with CLD. Biological aging clocks may serve as novel biomarkers to stratify early therapeutic interventions for CLD and potentially other chronic inflammatory diseases. Future research should include early-stage clinical trials using agents that target increased aging processes in patients with CLD, such as senolytic drugs.

### Supplementary information

Supplementary information accompanies the manuscript on the Experimental & Molecular Medicine website (http://www.nature.com/emm/).

### Supplementary information

Supplementary figures 1-8
