# Supplementary material for: Accelerated aging of skeletal muscle and the immune system in patients with chronic liver disease
Source: Exp Mol Med. 2024 Jul 18;56(7):1667–81. doi: 10.1038/s12276-024-01287-y (PMC11297261; doi:10.1038/s12276-024-01287-y)
Supplement: Supplementary file 1 — Supplementary figures 1-8 [file 12276_2024_1287_MOESM1_ESM.pdf]

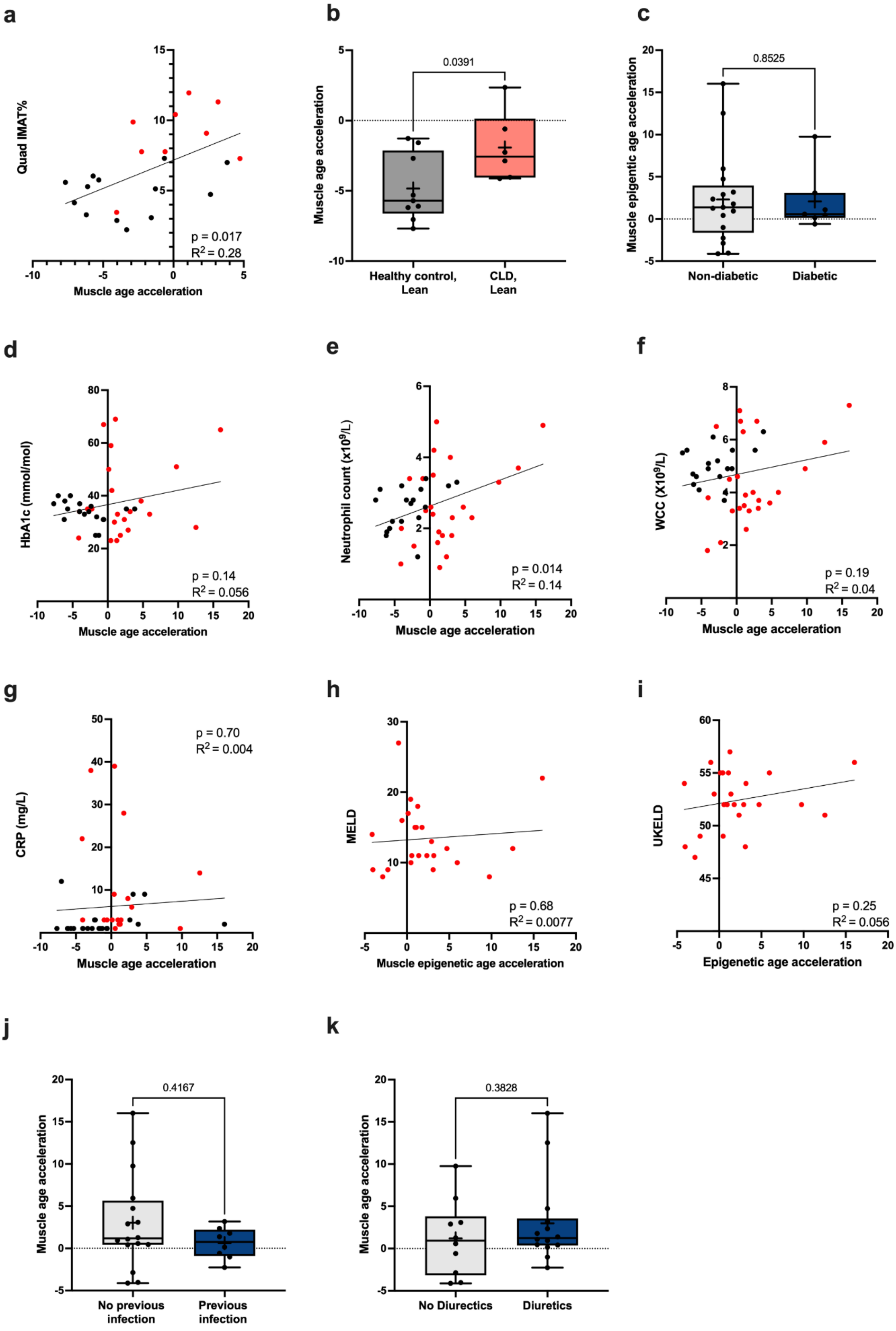

a
Quad IMAT%
Muscle age acceleration
p = 0.017
R2 = 0.28
b
Muscle age acceleration
0.0391
Healthy control, Lean
CLD, Lean
c
Muscle epigenetic age acceleration
0.8525
Non-diabetic
Diabetic
d
HbA1c (mmol/mol)
Muscle age acceleration
p = 0.14
R2 = 0.056
e
Neutrophil count (x109/L)
Muscle age acceleration
p = 0.014
R2 = 0.14
f
WCC (X109/L)
Muscle age acceleration
p = 0.19
R2 = 0.04
g
CRP (mg/L)
Muscle age acceleration
p = 0.70
R2 = 0.004
h
MELD
Muscle epigenetic age acceleration
p = 0.68
R2 = 0.0077
i
UKELD
Epigenetic age acceleration
p = 0.25
R2 = 0.056
j
Muscle age acceleration
0.4167
No previous infection
Previous infection
k
Muscle age acceleration
0.3828
No Diurectics
Diuretics

**Supplementary Fig. 1. Association of muscle Epigenetic age acceleration and clinical parameters**

**a.** Scatter plot displaying the association of muscle epigenetic age acceleration and quad IMAT in lean individuals only (BMI<25). **b.** Muscle epigenetic age acceleration in lean healthy and lean CLD patients only. **c.** Comparison of muscle age acceleration in CLD patients with or without diabetes. **d-i.** Scatter plots displaying the association of muscle epigenetic age acceleration and blood HbA1c, neutrophil count, white cell count, CRP, MELD score and UKELD score respectively. **j-k.** Comparison of muscle age acceleration in CLD patients with without previous spontaneous bacterial peritonitis infection or with and without prescribed diuretics respectively. Black symbols denote healthy control individuals, red symbols denote CLD patients. IMAT, intramuscular adipose tissue. CRP, C-reactive protein. WCC, white cell count. MELD, Model for End-Stage Liver Disease. UKELD, United Kingdom Model for End-Stage Liver

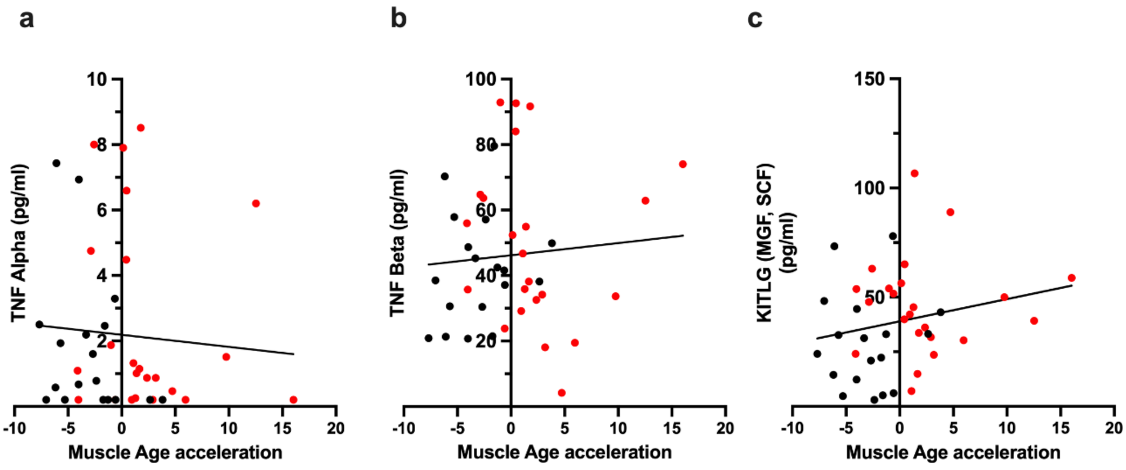


**Supplementary Fig. 2. Association of muscle epigenetic age acceleration and cytokine levels.**
Scatter plots displaying the association of muscle Epigenetic age acceleration associated with serum levels of **a.** TNF-alpha, **b.** TNF-beta and **c.** KITLG. Black symbols denote healthy control individuals, red symbols denote CLD patients.

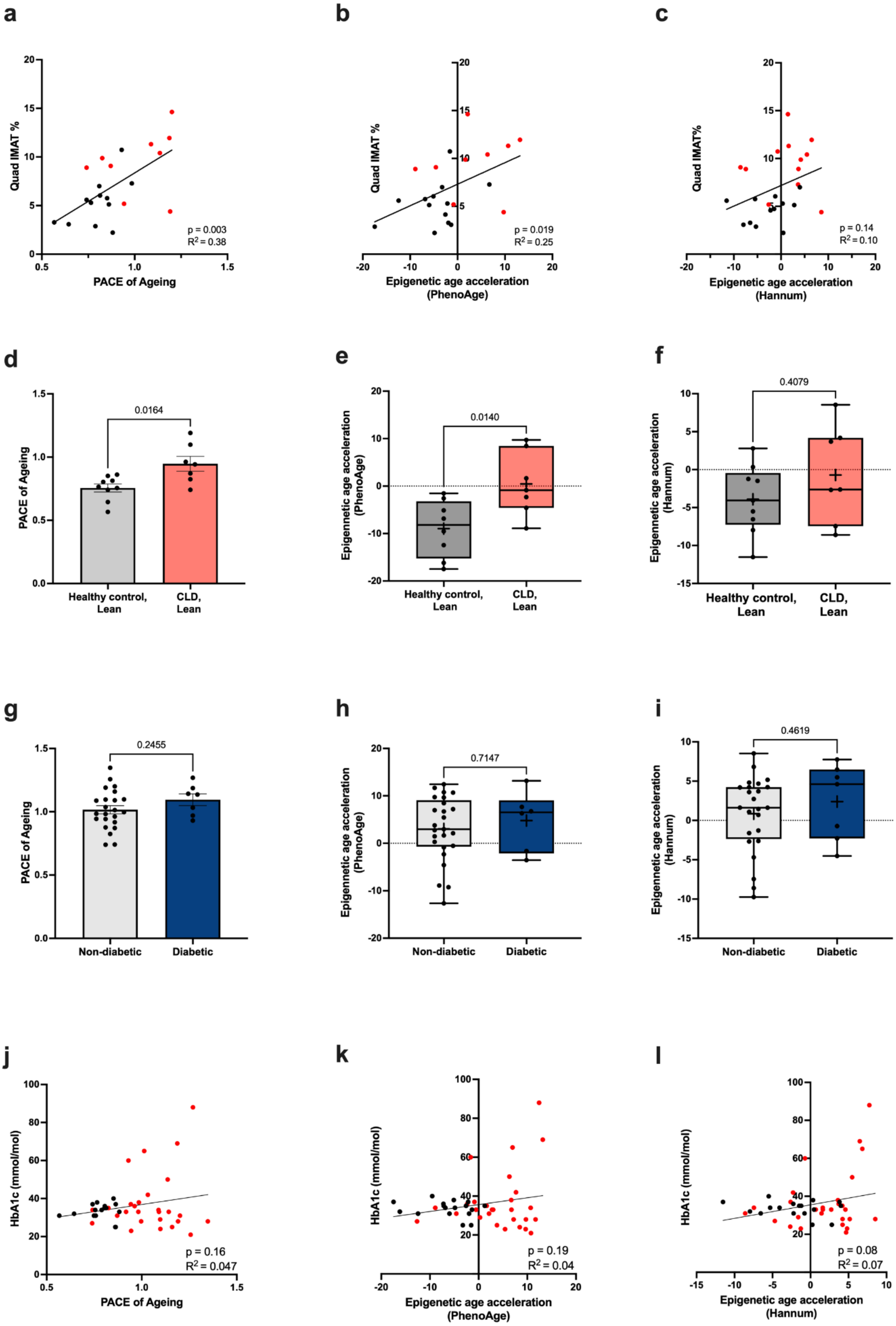

a
Quad IMAT %
PACE of Ageing
p = 0.003
$R^2$ = 0.38
b
Quad IMAT %
Epigenetic age acceleration (PhenoAge)
p = 0.019
$R^2$ = 0.25
c
Quad IMAT%
Epigenetic age acceleration (Hannum)
p = 0.14
$R^2$ = 0.10
d
PACE of Ageing
0.0164
Healthy control, Lean
CLD, Lean
e
Epigennetic age acceleration (PhenoAge)
0.0140
Healthy control, Lean
CLD, Lean
f
Epigennetic age acceleration (Hannum)
0.4079
Healthy control, Lean
CLD, Lean
g
PACE of Ageing
0.2455
Non-diabetic
Diabetic
h
Epigennetic age acceleration (PhenoAge)
0.7147
Non-diabetic
Diabetic
i
Epigennetic age acceleration (Hannum)
0.4619
Non-diabetic
Diabetic
j
HbA1c (mmol/mol)
PACE of Ageing
p = 0.16
$R^2$ = 0.047
k
HbA1c (mmol/mol)
Epigenetic age acceleration (PhenoAge)
p = 0.19
$R^2$ = 0.04
l
HbA1c (mmol/mol)
Epigenetic age acceleration (Hannum)
p = 0.08
$R^2$ = 0.07

**Supplementary Fig. 3. PBMC Epigenetic age acceleration and adiposity related parameters**

**a-c.** Scatter plots displaying the association of quad IMAT% with, PBMC PACE of Ageing, Phenoage acceleration and Hannum age acceleration respectively, in lean individuals only (BMI<25). **d-f.** PBMC PACE of Ageing, Phenoage acceleration and Hannum age acceleration respectively, in lean healthy controls compared to lean CLD patients. **g-i** PBMC PACE of Ageing, Phenoage acceleration and Hannum age acceleration respectively, in CLD patients with or without diabetes. **j-l** Scatter plots displaying the association of quad blood HbA1c with PBMC PACE of Ageing, Phenoage acceleration and Hannum age acceleration respectively. Black symbols denote healthy control individuals, red symbols denote CLD patients. BMI, body mass index. IMAT, intramuscular adipose tissue.

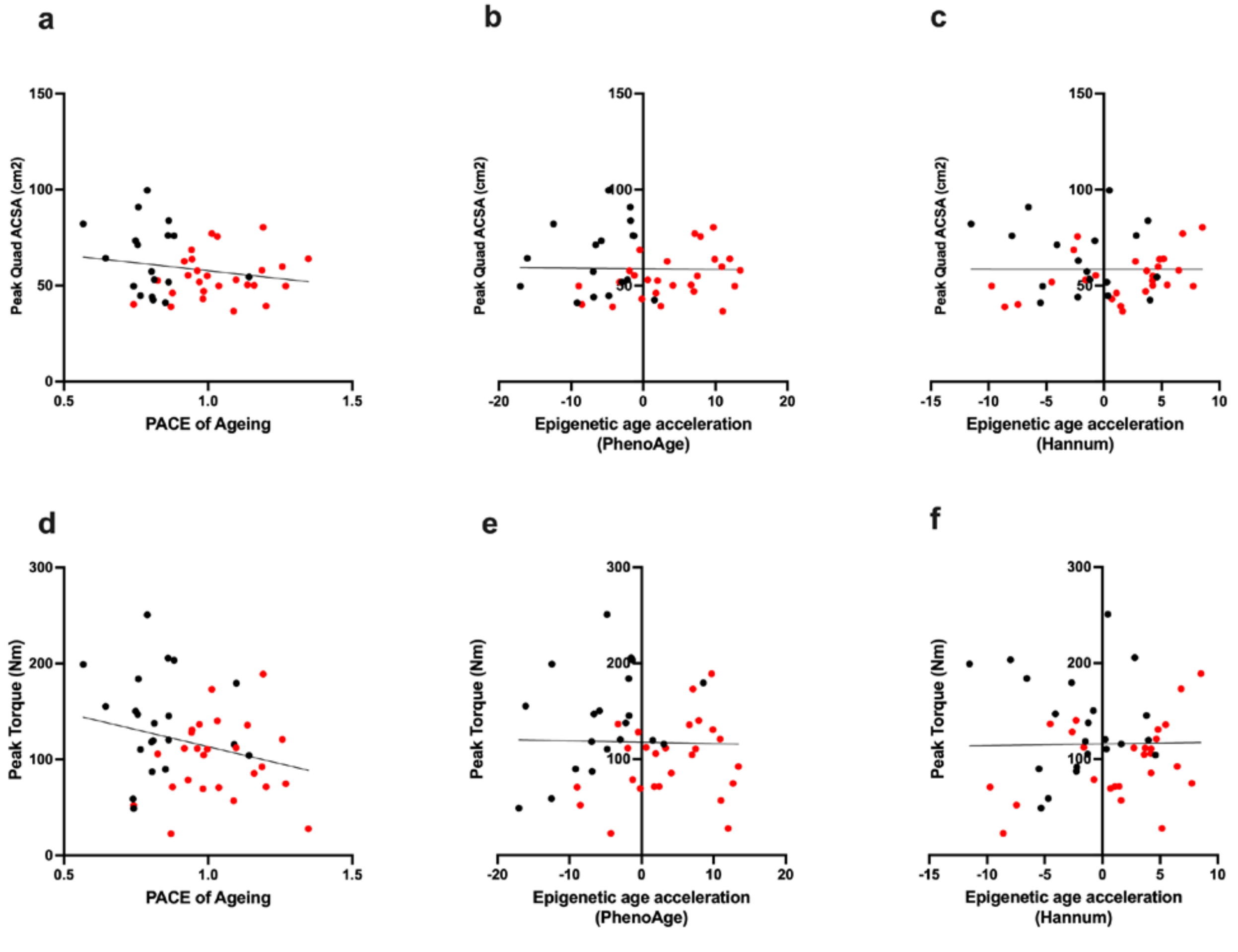


**Supplementary Fig. 4. Association of PBMC epigenetic age acceleration and measures of muscle mass and function**

Scatter plots displaying the association of peak quad ACSA and PBMC epigenetic ageing calculated by **a.** PACE of Ageing. **b.** PhenoAge acceleration. **c.** Hannum age acceleration. The association of quad peak torque and PBMC epigenetic ageing calculated by **d.** PACE of Ageing. **e.** PhenoAge acceleration. **f.** Hannum age acceleration. Black symbols denote healthy control individuals, red symbols denote CLD patients. ACSA, anatomical cross-sectional area.

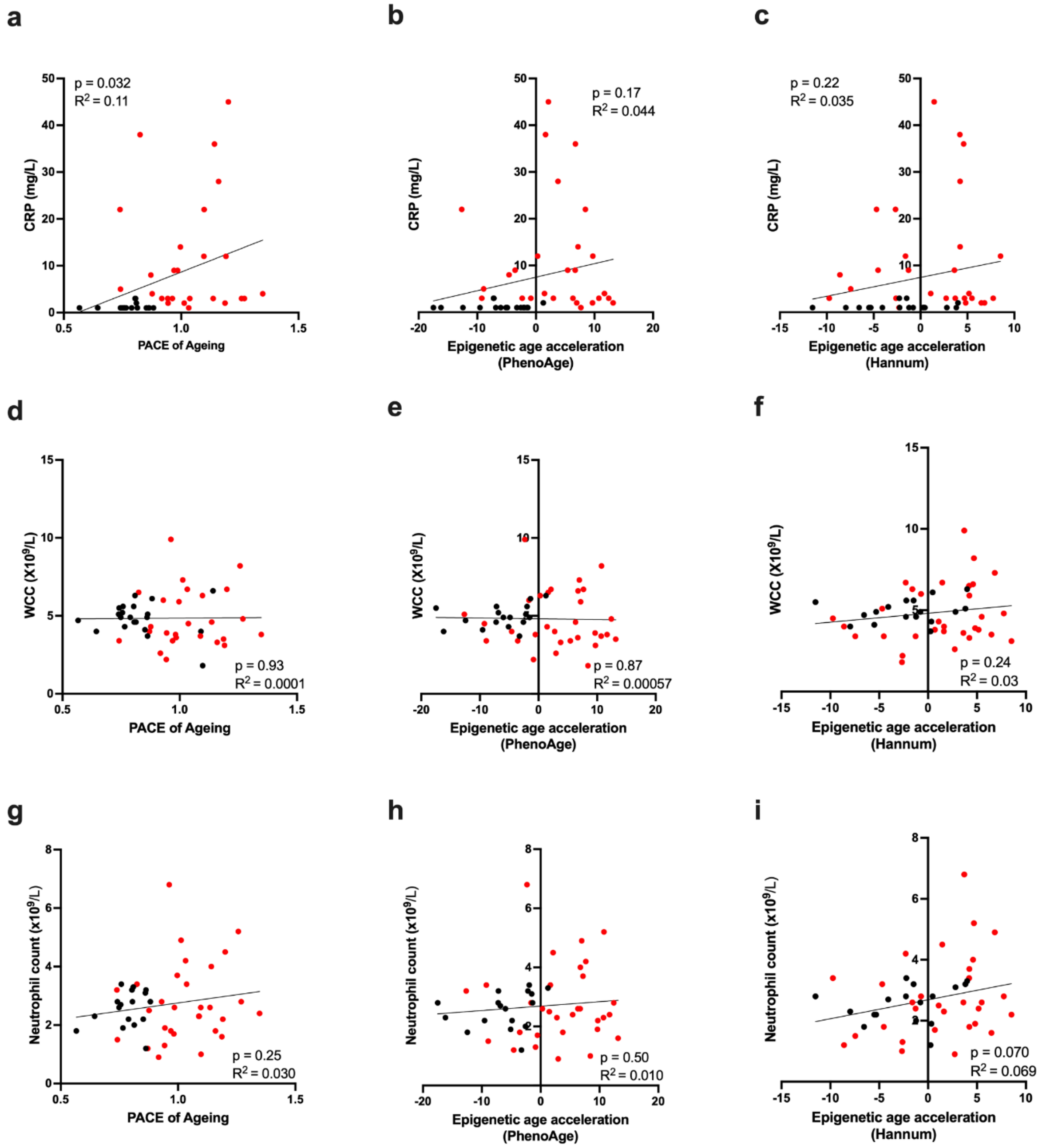


**Supplementary Fig. 5. Association of PBMC epigenetic age acceleration and blood markers**
**a-c.** Scatter plots displaying the association of CRP with PBMC PACE of Ageing, PhenoAge acceleration and Hannum age acceleration Respectively. **d-f.** Scatter plots displaying the association of WCC with PACE of Ageing, PhenoAge acceleration and Hannum age acceleration Respectively. **g-i**. Scatter plots displaying the association of neutrophil count with PBMC PACE of Ageing, PhenoAge acceleration and Hannum age acceleration respectively. Black symbols denote healthy control individuals, red symbols denote CLD patients. CRP, C-reactive protein. WCC, white cell count.

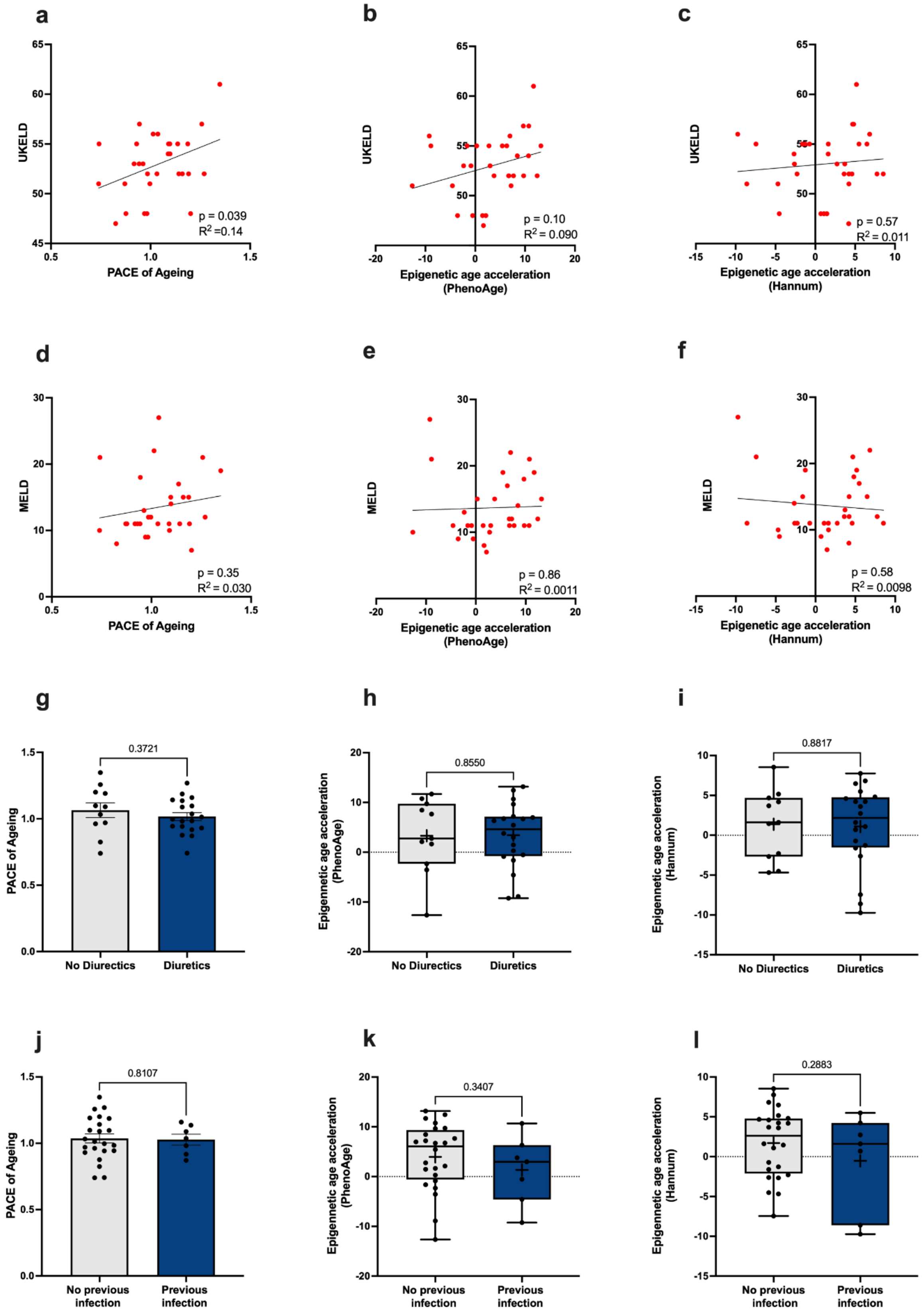

a
UKELD
PACE of Ageing
p = 0.039
R2 =0.14
b
UKELD
Epigenetic age acceleration
(PhenoAge)
p = 0.10
R2 = 0.090
c
UKELD
Epigenetic age acceleration
(Hannum)
p = 0.57
R2 = 0.011
d
MELD
PACE of Ageing
p = 0.35
R2 = 0.030
e
MELD
Epigenetic age acceleration
(PhenoAge)
p = 0.86
R2 = 0.0011
f
MELD
Epigenetic age acceleration
(Hannum)
p = 0.58
R2 = 0.0098
g
PACE of Ageing
0.3721
No Diurectics
Diuretics
h
Epigenetic age acceleration
(PhenoAge)
0.8550
No Diurectics
Diuretics
i
Epigenetic age acceleration
(Hannum)
0.8817
No Diurectics
Diuretics
j
PACE of Ageing
0.8107
No previous infection
Previous infection
k
Epigennetic age acceleration
(PhenoAge)
0.3407
No previous infection
Previous infection
l
Epigenetic age acceleration
(Hannum)
0.2883
No previous infection
Previous infection

**Supplementary Fig. 6. Association of PBMC epigenetic age acceleration and clinical parameters**
**a-c.** Scatter plots displaying the association of UKELD score and PBMC PACE of Ageing, Phenoage acceleration and Hannum age acceleration respectively. **d-f** Scatter plots displaying the association of MELD score and PBMC PACE of Ageing, Phenoage acceleration and Hannum age acceleration respectively. **g-i.** Comparison of PBMC PACE of Ageing, Phenoage acceleration and Hannum age acceleration respectively in CLD patients with or without prescribed use of diuretics. **j-l.** Comparison of PBMC PACE of Ageing, Phenoage acceleration and Hannum age acceleration respectively in CLD patients with or without prior spontaneous bacterial peritonitis infection. MELD, Model for End-Stage Liver Disease. UKELD, United Kingdom Model for End-Stage Liver Disease.

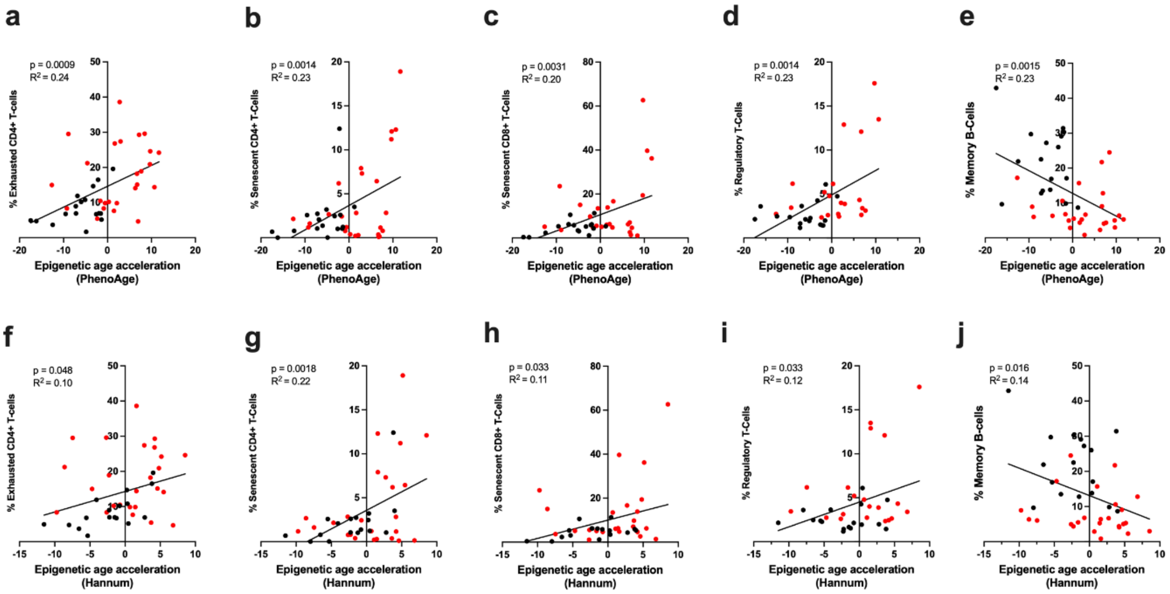


**Supplementary Fig. 7. Association of age-associated immune cell populations and PBMC epigenetic age acceleration.**

Scatter plots displaying the association of PBMC PhenoAge acceleration and **a**. % Exhausted T-cells. **b.** Senescent $CD4^+$ T-cells. **c.** % Senescent $CD8^+$ T-cells. **d. %** Regulatory T-cells. **e.** % Memory B-cells. Equivalent data for Hannum age acceleration is presented in **f-j**. Black symbols denote healthy control individuals, red symbols denote CLD patients.

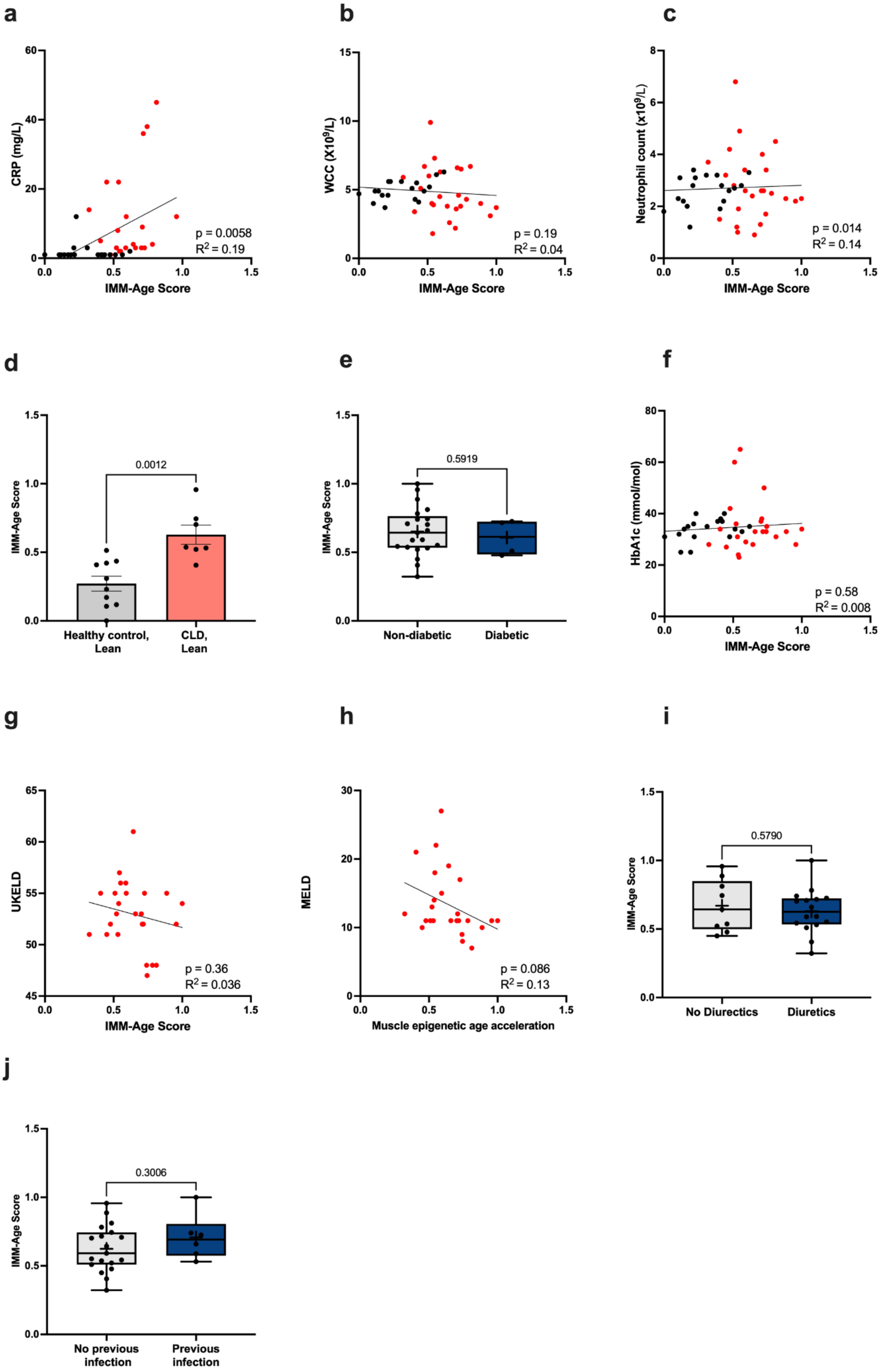
a
CRP (mg/L)
IMM-Age Score
p = 0.0058
R2 = 0.19
b
WCC (X10⁹/L)
IMM-Age Score
p = 0.19
R2 = 0.04
c
Neutrophil count (x10⁹/L)
IMM-Age Score
p = 0.014
R2 = 0.14
d
IMM-Age Score
0.0012
Healthy control, Lean
CLD, Lean
e
IMM-Age Score
0.5919
Non-diabetic
Diabetic
f
HbA1c (mmol/mol)
IMM-Age Score
p = 0.58
R2 = 0.008
g
UKELD
IMM-Age Score
p = 0.36
R2 = 0.036
h
MELD
Muscle epigenetic age acceleration
p = 0.086
R2 = 0.13
i
IMM-Age Score
0.5790
No Diurectics
Diuretics
j
IMM-Age Score
0.3006
No previous infection
Previous infection

**Supplementary Fig. 8. Association of PBMC IMM-Age and clinical parameters**

**a-c.** Scatter plots displaying the association of IMM-Age score with blood CRP, WCC and neutrophil counts respectively. **d.** Comparison of IMM-Age score in lean healthy control and CLD individuals (BMI<25). **e.** Comparison of IMM-Age score between diabetic and non-diabetic individuals with CLD. **f-h** Scatter plots displaying the association of IMM-Age score with blood HbA1c and UKELD and MELD scores respectively. **i.** Comparison of IMM-Age score between CLD patients with or without prescribed use of diuretics. **j.** Comparison of IMM-Age score between CLD patients with or without previous spontaneous bacterial peritonitis infection. Black symbols denote healthy control individuals, red symbols denote CLD patients. CRP, C-reactive protein. WCC, white cell count. MELD, Model for End-Stage Liver Disease. UKELD, United Kingdom Model for End-Stage Liver Disease.
